# Psychological Profile of Nurse Managers in the Post-COVID-19 Era: Implications for Nursing Leadership

**DOI:** 10.1155/2024/8428954

**Published:** 2024-06-14

**Authors:** Majed Mowanes Alruwaili

**Affiliations:** Nursing Administration and Education Department, College of Nursing, Jouf University, Sakaka 72388, Saudi Arabia

## Abstract

**Aim:**

This study examined the mental health of nurse managers in the Kingdom of Saudi Arabia.

**Background:**

The COVID-19 pandemic has affected the physical and mental health of senior nurses, including effects of shortages of staff and medical supplies. However, no study has examined this topic among nurse managers in the Kingdom of Saudi Arabia despite their exposure to mental stress during the pandemic.

**Methods:**

A cross-sectional design was used to investigate the levels of depression, anxiety, stress, and general psychological distress among nurse managers in Saudi Arabia. Data were collected August 2023 to February 2024. The main tool was the reliable and validated Arabic translation of the Depression, Anxiety, and Stress Scale (DASS-21). Data collection was performed using an online platform. IBM SPSS software was used for data analysis. The data was analysed using multiple regression to examine the relationship between the dependent (outcome) and independent (predictor) variables. A significant *p*-value was set at 0.05.

**Results:**

Stress and general psychological distress were the most common problems among nurse managers in the post-COVID-19 era. Individual educational attainment was the only significant predictor of anxiety, stress, and general psychological distress. Moreover, the nationalities of nurse managers were correlated with stress outcomes.

**Conclusions:**

Nursing managers are very likely to suffer from stress and general mental health problems in cases of exposure to crises. They may find the results of this study useful in understanding the factors that may play a role in the development of mental health problems during clinical work. Different strategies can be considered to alleviate depression, anxiety, and stress among managers, including the proper delegation of tasks. Top-level management and healthcare stakeholders should give special considerations to the nationality and education level upon selecting nurse mangers at different levels. *Implications in Nursing Management*. Policy makers involved in planning care for healthcare professionals may find this study valuable in planning for future pandemics by developing a strategy that could reduce stress and psychological distress.

## 1. Introduction

The role of care managers has become increasingly important since the advent of COVID-19 as competent care management can mitigate many of the problems that nurses face [1]. Hospital management by nurse managers has been found to be associated with staff shortages, crisis management, and related psychological problems [2]. Several factors put the physical and mental health of caregivers at risk from COVID-19, including shortages of staff and medical supplies, a lack of personal protective equipment (PPE), and limited hospital beds [3]. Nurse managers also struggle with physical and mental health problems [1]. COVID-19 has presented a challenge to hospital managers as increasing demand for care was accompanied by workforce shortages and crisis management. The mental health of nurse managers can be compromised by demands of providing emotional support, managing medical supply and Personal Protective Equipment (PPE) inventories, and supporting team members during pandemics [4–6]. In addition, the lack of previous experience in dealing with pandemics and the lack of effective preparation by the respective authorities or policy makers could also contribute to the management challenges [1, 2]. The reasons given for the scale of the problem were the complex and expensive diagnostic procedures and therapeutic equipment and application methods, combined with the need for more staff and the shortage of hospital beds, as well as disproportionate management for the allocation of resources and the lack of a standard program for the preparation and response phase, alongside inadequate staff training and practice and the varying experience of health managers, which made the situation more difficult for nurse managers and their subordinates [2, 3, 7].

To date, no study has examined the mental health of nurse managers in the Kingdom of Saudi Arabia (KSA) in this context, despite their exposure to psychological stress during the pandemic. The pandemic lasted for more than three years, and professionals have continuously taken precautions to contain it. It is believed that it is appropriate to assess the mental health of nurse managers since they have continuously provided managerial guidance and control during and after the pandemic, and the resulting stress can increase the risk of mental health problems. Hence the need for this study, which could potentially reveal the extent of the problem and possible measures to mitigate it in the clinical setting.

The COVID-19 pandemic has had a significant impact on the mental health of adults and children alike [8]. Nearly 50% of the respondents to a US survey had recently experienced anxiety or sadness, and 10% felt that their mental health needs were not being met [8]. The rates of anxiety, depression, and substance-use disorders have increased since the epidemic began [9]. People with mental illnesses or disorders that are worsened by COVID-19 were found to be more likely to die than people without mental illnesses [8]. Mental illness has become an important research topic, and researchers are developing strategies to diagnose, prevent, and treat them [8, 9].

Evidence suggests that infected individuals are more likely to develop mental illness or disorders, including posttraumatic stress disorder [9]. The symptoms associated with long COVID-19 can affect brain functioning and mental state [8, 9]. In particular, the presence of COVID-19 may be associated with cognitive and attention deficits (brain fog), anxiety, depression, psychosis, seizures, and suicidality [8].

There is a potential threat to the health and wellbeing of leaders of hospitals and healthcare organizations (including leaders in nursing) due to increasing demands for restructuring of infection control systems [8]. Therefore, it is necessary to examine the psychological state of leaders in nursing in the post-COVID-19 era. Such information could help in the future design and development of health system policies that address staff wellbeing. The study objectives were to investigate the levels of depression, anxiety, stress, and general psychological distress among nurse managers in the post-COVID-19 pandemic era and to explore factors that affect their psychological profiles.

## 2. Methods

### 2.1. Study Design, Population, and Settings

This study used a cross-sectional survey design. Participants were recruited from June to November 2023 using convenience sampling. The software G^*∗*^Power Online 3.1.9.4 was used to determine the sample size needed based on a previous study investigating ethical leadership of nurses with a moderate effect size of 0.65 [10]. With a power of 80% (0.8) and a significance level of 0.5, the required sample size was 76 participants. Taking into account the possibility of 20% attrition after 2 months, the total sample size for the study groups was 92 participants. The criteria for participation in the study were age >18 years, employment in a public hospital as a nurse manager (e.g., nursing directors, heads/supervisors of nursing departments, and ward managers), and the ability to speak Arabic or English. The participants were recruited from all regions of the Kingdom of Saudi Arabia.

### 2.2. Measures

A wide range of sociodemographic information was collected from the participants, such as age, sex, marital status, employment, and level of education. Data were collected online by a trained research assistant. Informed consent forms were provided to all eligible participants after an explanation of the study procedure was given.

The Arabic version of the Depression, Anxiety, and Stress Scale (DASS-21) was used to assess symptoms of depression, anxiety, stress, and general psychological distress [11–13].

The DASS-21 test contains 21 items and three subscales: depression, anxiety, and stress [11, 14]. With the exception of general psychological distress, which comprises all 21 items, all three subscales had seven items each. Each construct is measured with four-point Likert scale (0–3) representing frequency, where 0 represents “never,” 1 represents “sometimes,” 2 represents “often,” and 3 represents “almost always.” Based on the total number of items on each subscale, the total subscale scores range from 0 to 21 [15]. Multiplying each of the subscale scores by 2 gives a score similar to that of the DASS-42 [15]. Depressive symptoms were considered normal for scores of 0–9, moderate for scores of 10–13, severe for scores of 14–20, and extremely severe for scores of 20+. The stress subscale ranges are 0–14, 15–18, 19–25, 26–33, and 34+, with 0–14 indicating normal symptoms, 15–18 indicating mild symptoms, 19–25 indicating moderate symptoms, and 26–33 indicating severe symptoms [16]. Anxiety scores of 0–7 indicate fairly normal anxiety, 8-9 indicate mild anxiety symptoms, 10–14 indicate moderate anxiety symptoms, and 15–19 indicate severe anxiety symptoms [16].

The instrument has been translated into over fifty different languages and has been shown to be valid for assessing the constructs of depression, anxiety, and stress [17]. However, the construct of general psychological distress was also reported [18]. This confirms the existence of a two-factor model in its construct. DASS-21 has been psychometrically tested in diverse populations, including various racial groups with internal consistency ranging from 0.74 to 0.84 [19], clinical groups, and communities, with equally acceptable concurrent validity and internal consistency reliability [20], and nonclinical samples of adults (0.80–0.91) for the three constructs of depression, anxiety, and stress [17]. Similarly, it was found to have internal consistencies of 0.72, 0.72, and 0.76 for depression, stress, and anxiety among the Internally Displaced Persons [18]. For this study, the Arabic and English versions were used. Finally, the four respective constructs in this cohort had an internal consistency of 0.82, 0.72, 0.61, and 0.50 for general psychological distress, stress, depression, and anxiety, respectively.

### 2.3. Ethical Considerations

Ethical approval was obtained from the Institutional Review Board of Jouf University with reference number 6-11-44. The study was carried out in accordance with the Declaration of Helsinki and in strict compliance with its principles. All subjects participating were provided with the necessary information about the study to provide informed consent and had the right to terminate participation at any time without penalty.

### 2.4. Data Analysis

The statistical software IBM SPSS version 20 (IBM Corp., Armonk, NY, USA) was used for data analysis. The level of significance was set at *p* < 0.05. Age, sex, marital status, employment status, and educational status were analysed using descriptive statistics of frequencies and percentages. Multiple regressions using the standard method were used to evaluate the predictive relationship between the independent variables (age, sex, marital status, education, and employment) and the dependent variables (symptoms of depression, anxiety, stress, and general psychological distress). The analysis was carried out based on the domains of DASS-21, so four different regression analyses were carried out and are presented as models 1, 2, 3, and 4. The results are presented as unstandardized coefficients (B) with standard error (SE), confidence intervals, *R*^2^ values, and significance values.

Due to the categorical nature of marital status and employment, dummy variables were created. Age and education were treated as continuous variables because they were categorical variables with five levels of ordering. The three outcome variables of depression, anxiety, and stress symptoms were treated as categorical variables. Preliminary analyses were conducted to ensure the assumptions of linearity, homoscedasticity, and normality using scatter plots and residuals [21].

The Q-Q plot was examined for kurtosis and skewness to determine whether the data were normally distributed. A rectangular distribution of residuals against the predicted value was used to assess the assumption of homoscedasticity [22]. Several other checks were conducted, including the adequacy of the samples, the assumption of singularity, multicollinearity, and correlation examination.

Data cleaning was conducted for the entered data to identify any discrepancies between them and the raw data and ensure the accuracy of the analysis [23]. The data were checked against a printout for five datasets and were compared with descriptions when systematic errors were detected to identify missing data. The mean, standard deviation, frequencies, minima, and maxima were used to determine extreme values for continuous variables. Incorrect data entries were indicated by unexpectedly low values in the minimum column [24]. The questionnaires were kept confidential in one drive.

## 3. Results

The majority of the study population was between 35 and 44 years old. A bachelor's degree was the most widely reported qualification of the nurse managers. High proportions of the participants were Saudi nationals (93.6%), married (77.1%), and employed full time (92.7%) (Table 1).

As shown in Table 2, multiple regression analysis was utilized to assess levels of anxiety, stress, depression, and general psychological distress after controlling for age, sex, marital status, education, and employment status. Models 1, 2, 3, and 4 showed variance of 8.0, 3.8, 9.4, and 8.7 for anxiety, depression, stress, and general psychological distress, respectively. However, only stress and general psychological distress reached statistical significance. The only variable that made a significant contribution to the development of anxiety was the level of education (*B* = −1.60, SE 0.62, 95% confidence interval (CI) −2.83, −0.38, *p* < 0.05). Individual nationality (*B* = 2.48, SE 1.07, 95% CI 0.35, 4.61) and educational level (*B* = −1.78, SE 0.78, 95% CI −3.32, −0.25, *p* < 0.05) were predictors of stress. Finally, education had predictive power for general psychological distress (*B* −4.83, SE 1.79, 95% CI −8.38, 1.28, *p* < 0.05).

## 4. Discussion

To our knowledge, this is the first study to look at the psychological profile of nurse managers in the post-COVID-19 era in KSA. Factors that can contribute to the development of mental health problems among nurse managers were also examined. This study shows that stress and general psychological distress appear to be the most common problems among nurse managers in the healthcare sector. The individual level of education was the only significant predictor of anxiety, stress, and general psychological distress. It was also found that the nationality of the nurse managers correlated with stress.

Corroborating with similar studies, evidence have shown that healthcare professionals suffer from a range of mental health problems due to a lack of equipment and work overload, which may have an impact on the quality of care [25, 26]. This study found that educational level and individual nationality contribute to the development of stress, while education alone has predictive power for general psychological stress. Education has been shown in the literature to be a significant predictor of mental health (depression, anxiety, and stress) [25, 27]. In other words, marital status, gender, and monthly working hours were significant predictors of mental health [25]; however, they were not in this study. Therefore, further quantification is needed.

Stress has been reported to be a common problem among nurses and may be due to staff shortages, poor working conditions, inadequate management support, and high workload [28]. Furthermore, an association has been found among nurses between COVID-19-related work stressors and psychological distress [29–31]. Studies have also reported that nurses with lower educational qualifications are more stressed than those with higher qualifications [32]. The literature also shows that a lower level of psychological stress can be correlated with a higher level of education, and vice versa [33]. Anxiety is also widespread among nurses with lower academic qualifications [34].

The emergence of COVID-19 has posed a threat to the health system and led to structural, environmental, and administrative changes or requirements that have continued into the post-pandemic period. Therefore, the stress and general psychological distress in this study are not unusual. Evidence has shown that chronic stressors have affected people worldwide and across all sections of society, and without a doubt, the COVID-19 pandemic has been a contributing factor [35]. As a result, it may have provoked a crisis of unprecedented proportions in public mental health [35, 36].

The COVID-19 interventions have required all people to stay indoors and maintain physical distance in any situation (including in hospitals) or when they are out for any purpose [36]. The prevalence of common mental disorders such as depression, anxiety, and stress has been expected to increase in the post-COVID-19 pandemic period due to the long-term effects of restrictive measures such as quarantine and social distancing, as well as the socioeconomic consequences [37]. In an event of such magnitude as the pandemic, the impact on mental health can be long-lasting [37].

Physical and emotional exhaustion and fear of infection among nurses further aggravate their fears [38]. There has been a huge impact on people's mental health as well as on all current activities as a result of the aforementioned interventions [36]. In particular, positive mental health outcomes were associated with organizational support, readiness, safety, and materials and resources [38]. Evidence has shown that the health consequences of nurse management may be similar to those faced by front-line nurses [38].

The COVID-19 pandemic posed a resource challenge for many institutions, and nurse managers had the responsibility of managing human and equipment resources [38]. Nursing care managers in particular are responsible for making decisions regarding staff and patients, planning care, and supervising the nurses who provide this care [38]. They may have been impacted by uncertainty related to COVID-19 management because practical measures were constantly changing.

In KSA, nurse managers comprise both local people and expatriates. However, a study has shown that occupational stress is widespread among expatriates [39]. Therefore, it should not be surprising that stress is reportedly associated with nationality. Furthermore, psychological distress, anxiety, and stress are significantly correlated with education. This result is in line with the study by Golubic and colleagues [32], who found that nurses with a lower level of education experienced more stress at work compared to those with a college degree. A lower level of education could also be correlated with the development of anxiety, and according to Bjelland et al. [40], a higher level of education appears to protect against anxiety.

Similarly, studies have shown that there is a positive correlation between higher education and lower psychological stress [33]. The level of education alone could be a predictor of general psychological distress. Therefore, it is not surprising that general psychological distress is significantly correlated with educational levels in the present study. Notably, only 12.8% of the study participants had post-graduate degrees.

### 4.1. Limitations

One of the limitations of this study is that the sample size was small, which could limit the generalizability of the results. Furthermore, the cross-sectional nature of this study limits our ability to infer causality and provides only a partial picture of the situation. As data were collected using a self-report questionnaire, the results cannot be interpreted with certainty. Therefore, an in-depth study using a mixed methods design that incorporate a qualitative component should be carried out to find out more about the mental health problems associated with COVID-19.

## 5. Conclusion

Although this study had limitations, it contributes to our understanding of mental health outcomes. Stress and general psychological distress appear to be the main mental health problems among nurse managers. It was also found that individual nationality and level of education play important roles in the development of anxiety, stress, and general psychological distress. The results of this study can raise nurse leaders' awareness of the levels of anxiety, stress, and depression and help to provide psychological support programs to improve nurses' mental health during the pandemic. Therefore, policy makers should develop a blueprint for monitoring the mental health of facility managers in public health facilities. Furthermore, healthcare institutions should ensure that more professionals with higher education be appointed as care managers. Managers should delegate tasks to junior staff as a strategy to deal with some work-related stressors.

## Figures and Tables

**Table 1:** Demographics.

No	Variables	Frequencies (%)	M (SD)
1	*Age*		3.04 (0.77)
	25–34	30 (27.5)	
	35–44 years	45 (41.3)	
	45–54 years	34 (31.2)	
2	*Educational levels*		1.13 (0.34)
	Degree (Bachelor's)	95 (87.2)	
	Postgraduate (masters)	14 (12.8)	
3	*Marital status*		1.92 (0.72)
	Single	21 (19.3)	
	Married	84 (77.1)	
	Divorced	4 (3.7)	
4	*Employment status*		1.17 (0.64)
	Full-time	101 (92.7)	
	Self-employed	5 (4.6)	
	Locum contract	3 (2.8)	
5	*Nationality*		1.06 (0.25)
	Saudi	102 (93.6)	
	Non-Saudi	7 (6.4)	

**Table 2:** Summary of the multiple regression analysis to predict the level of anxiety, stress, depression, and general psychological distress with age, sex, nationality, education, and employment status.

Variable	*B*	SE *B*	95% CI	*R* ^2^	*p*.value
Model 1 (DASS-21-anxiety subscale)				0.080	0.066
Constant	11.72	1.19	9.37, 14.08		<0.001
Nationality	1.35	0.86	−0.35, 30.04		0.12
Marital status	0.15	0.54	−0.91, 1.21		0.78
Education	−1.60	0.62	−2.83, −0.38		<0.05
Employment	−0.150	0.80	−1.74, 1.44		0.85
Model 2 (DASS-21-depression subscale)				0.038	0.395
Constant	12.44	1.46	9.55, 15.33		<0.001
Nationality	0.224	1.05	−1.86, 2.30		0.83
Marital status	−0.04	0.66	−1.34, 1.27		0.96
Education	−1.444	0.756	−2.94, 0.06		0.06
Employment	0.612	0.983	−1.34, 2.56		0.54
Model 3 (DASS-21-stress subscale)				0.094	<0.05
Constant	10.64	1.49	7.68, 13.60		<0.001
Nationality	2.48	1.07	0.35, 4.61		<0.05
Marital status	0.67	0.67	−0.67, 2.0		0.32
Education	−1.78	0.78	−3.32, −0.25		<0.05
Employment	0.04	1.01	−1.96, 2.03		0.97
Model 4 (DASS-21-general psychological distress)				0.087	<0.05
Constant	34.81	3.45	27.97, 41.64		<0.001
Nationality	4.05	2.48	−0.87, 8.97		0.11
Marital status	0.78	1.55	−2.30, 3.87		0.62
Education	−4.83	1.79	−8.38, 1.28		<0.05
Employment	0.50	2.32	−4.11, 5.11		0.83

*Note*. CI: confidence interval; *B* = unstandardized beta; SE *B*: standard error of beta. (*N* = 109).

## Data Availability

Data for this study are available from the corresponding author upon reasonable request.

## Additional Points

*Copyright and Permissions*. The DASS questionnaire is public domain and so permission is not needed to use it according to the developers available at https://www2.psy.unsw.edu.au/groups/dass/DASSFAQ.htm#_3.__How_do_I_get_permission_to_use.

## References

[1] Aydogdu A. L. F. (2022). Challenges faced by nurse managers during the COVID-19 pandemic: an integrative review. *Journal of Research in Nursing*.

[2] Gab Allah A. R. (2021). Challenges facing nurse managers during and beyond COVID‐19 pandemic in relation to perceived organizational support. *Nursing Forum*.

[3] Sun N., Wei L., Shi S. (2020). A qualitative study on the psychological experience of caregivers of COVID-19 patients. *American Journal of Infection Control*.

[4] Ilo (OrganizaciónInternacionaldelTrabajo–Oit) (2020). *Cinco Formas de Proteger al Personal de Salud Durante la Crisis del COVID-19*.

[5] Raso R. (2020). Leadership in a pandemic: pressing the reset button. *Nursing Management*.

[6] Rodrigues N. H., da Silva L. G. A. (2020). Gestão da pandemiacoronavírusem um hospital: relato de experiênciaprofissional/Management of the coronavirus pandemic in a hospital: professional experience report. *Journal of Nursing and health*.

[7] American Hospital Association (2021). *Data Brief: Health Care Workforce Challenges Threaten Hospitals’ Ability to Care for Patients*.

[8] Marroquín B., Vine V., Morgan R. (2020). Mental health during the COVID-19 pandemic: effects of stay-at-home policies, social distancing behavior, and social resources. *Psychiatry Research*.

[9] Kumar A., Nayar K. R. (2021). COVID 19 and its mental health consequences. *Journal of Mental Health*.

[10] Kim S., Jeong S. H., Seo M. H. (2022). Nurses’ ethical leadership and related outcome variables: systematic review and meta‐analysis. *Journal of Nursing Management*.

[11] Henry J. D., Crawford J. R. (2005). The short‐form version of the Depression Anxiety Stress Scales (DASS‐21): construct validity and normative data in a large non‐clinical sample. *British Journal of Clinical Psychology*.

[12] Osman A., Wong J. L., Bagge C. L., Freedenthal S., Gutierrez P. M., Lozano G. (2012). The depression anxiety stress scales—21 (DASS‐21): further examination of dimensions, scale reliability, and correlates. *The Journal of Clinical Psychology*.

[13] Bottesi G., Ghisi M., Altoè G., Conforti E., Melli G., Sica C. (2015). The Italian version of the Depression Anxiety Stress Scales-21: factor structure and psychometric properties on community and clinical samples. *Comprehensive Psychiatry*.

[14] Oei T. P. S., Sawang S., Goh Y. W., Mukhtar F. (2013). Using the depression anxiety stress scale 21 (DASS‐21) across cultures. *International Journal of Psychology*.

[15] Beaufort I. N., De Weert-Van Oene G., Buwalda V. A., de Leeuw J., Goudriaan A. E. J. E. a. r. (2017). The depression, anxiety and stress scale (DASS-21) as a screener for depression in substance use disorder inpatients: a pilot study. *European Addiction Research*.

[16] Gomez R., Summers M., Summers A., Wolf A., Summers J. J. (2014). Depression Anxiety Stress Scales-21: factor structure and test-retest invariance, and temporal stability and uniqueness of latent factors in older adults. *Journal of Psychopathology and Behavioral Assessment*.

[17] Sinclair S. J., Siefert C. J., Slavin-Mulford J. M., Stein M. B., Renna M., Blais M. A. (2012). Psychometric evaluation and normative data for the depression, anxiety, and stress scales-21 (DASS-21) in a nonclinical sample of US adults. *Evaluation and the Health Professions*.

[18] Salihu D., Kwan R. Y. C., Wong E. M. L., Leung D. Y. P. Translation, cross-cultural adaption and psychometric properties of the Hausa version of the depression anxiety stress scale-21 (DASS-21) in the internally displaced persons in Africa.

[19] Norton P. J. (2007). Depression anxiety and stress scales (DASS-21): psychometric analysis across four racial groups. *Anxiety, Stress and Coping*.

[20] Antony M. M., Bieling P. J., Cox B. J., Enns M. W., Swinson R. P. (1998). Psychometric properties of the 42-item and 21-item versions of the depression anxiety stress scales in clinical groups and a community sample. *Psychological Assessment*.

[21] Pallant J. (2011). *Survival Manual. A Step by Step Guide to Data Analysis Using SPSS 4*.

[22] Tabachnick B. G., Fidell L. S., Ullman J. B. (2007). *Using Multivariate Statistics*.

[23] Portney L. G., Watkins M. P. (2009). *Foundations of Clinical Research: Applications to Practice*.

[24] Pallant J. (2020). *SPSS Survival Manual: A Step by Step Guide to Data Analysis Using IBM SPSS*.

[25] Middleton R., Loveday C., Hobbs C. (2021). The COVID-19 pandemic–A focus on nurse managers’ mental health, coping behaviours and organisational commitment. *Collegian*.

[26] Hadian M., Jabbari A., Abdollahi M., Hosseini E., Sheikhbardsiri H. (2022). Explore pre-hospital emergency challenges in the face of the COVID-19 pandemic: a quality content analysis in the Iranian context. *Frontiers in Public Health*.

[27] Beyramijam M., Sheikhbardsiri H., Doustmohammadi M., Afshar P., Heidarijamebozorgi M., Khankeh H. (2021). Anxiety, stress and depression levels among nurses of educational hospitals in Iran: time of performing nursing care for suspected and confirmed COVID-19 patients. *Journal of Education and Health Promotion*.

[28] Ofei A. M., Paarima Y., Barnes T., Kwashie A. A. (2019). Stress and coping strategies among nurse managers. *Journal of Nursing Education and Practice*.

[29] Shahrour G., Dardas L. A. (2020). Acute stress disorder, coping self‐efficacy and subsequent psychological distress among nurses amid COVID‐19. *Journal of Nursing Management*.

[30] Hamama L., Marey‐Sarwan I., Hamama‐Raz Y., Nakad B., Asadi A. (2022). Psychological distress and perceived job stressors among hospital nurses and physicians during the COVID‐19 outbreak. *Journal of Advanced Nursing*.

[31] Sheikhbardsiri H., Tavan A., Afshar P. J., Salahi S., Heidari-Jamebozorgi M. (2022). Investigating the burden of disease dimensions (time-dependent, developmental, physical, social and emotional) among family caregivers with COVID-19 patients in Iran. *BMC primary care*.

[32] Golubic R., Milosevic M., Knezevic B., Mustajbegovic J. (2009). Work‐related stress, education and work ability among hospital nurses. *Journal of Advanced Nursing*.

[33] Brännlund A., Hammarström A. (2014). Higher education and psychological distress: a 27-year prospective cohort study in Sweden. *Scandinavian Journal of Public Health*.

[34] Shajan A., Nisha C. (2019). Anxiety and Depression among nurses working in a tertiary care hospital in South India. *International Journal of Advances in Medicine*.

[35] Pfeifer L. S., Heyers K., Ocklenburg S., Wolf O. T. (2021). Stress research during the COVID-19 pandemic and beyond. *Neuroscience and Biobehavioral Reviews*.

[36] Lakhan R., Agrawal A., Sharma M. (2020). Prevalence of depression, anxiety, and stress during COVID-19 pandemic. *Journal of Neurosciences in Rural Practice*.

[37] Kathirvel N. (2020). Post COVID-19 pandemic mental health challenges. *Asian journal of psychiatry*.

[38] White J. H. (2021). A phenomenological study of nurse managers’ and assistant nurse managers’ experiences during the COVID‐19 pandemic in the United States. *Journal of Nursing Management*.

[39] Doki S., Sasahara S., Matsuzaki I. (2018). Stress of working abroad: a systematic review. *International Archives of Occupational and Environmental Health*.

[40] Bjelland I., Krokstad S., Mykletun A., Dahl A. A., Tell G. S., Tambs K. (2008). Does a higher educational level protect against anxiety and depression? The HUNT study. *Social Science and Medicine*.

